# Supplementary material for: CMAPLE: Efficient Phylogenetic Inference in the Pandemic Era
Source: Mol Biol Evol. 2024 Jun 27;41(7):msae134. doi: 10.1093/molbev/msae134 (PMC11232695; doi:10.1093/molbev/msae134)
Supplement: msae134_Supplementary_Data [file msae134_supplementary_data.pdf]

SUPPLEMENTARY MATERIAL FOR

CMAPLE: efficient phylogenetic inference in the pandemic era

Nhan Ly-Trong, Chris Bielow, Nicola De Maio, Bui Quang Minh

Table S1. Runtimes, peak memory usage, and log-likelihoods of the trees inferred by four tools, CMAPLE, MAPLE, FastTree 2, and IQ-TREE 2, testing on 5K, 10K, 50K, 100K, and 200K SARS-CoV-2 sequences. IQ-TREE 2 and FastTree 2 were not run on 50K, 100K and 200K due to excessive runtimes. Each test was run three times, and the average results were reported. The best values among the four methods are in **boldface**.

#sequences	Software	Log-Likelihood	Difference from the best	Runtimes (hours)	Ratio to the shortest runtime	Peak memory usage (GB)	Ratio to the lowest peak memory usage
**5K**	CMAPLE	**-240,820**	0	**0.049**	1	**0.129**	1
	MAPLE	**-240,820**	0	0.183	3.735	0.525	4.07
	FastTree 2	-241,748	-927	1.436	29.306	7.787	60.364
	IQ-TREE 2	-241,739	-919	12.398	253.02	4.813	37.31
**10K**	CMAPLE	**-391,549**	0	**0.135**	1	**0.244**	1
	MAPLE	-391,590	-41	0.563	4.17	0.956	3.918
	FastTree 2	-393,393	-1,844	3.049	22.585	15.22	62.377
	IQ-TREE 2	-393,492	-1,944	40.488	299.911	12.542	51.402
**50K**	CMAPLE	**-1,274,343**	0	**2.036**	1	**1.056**	1
	MAPLE	-1,274,367	-24	6.412	3.149	3.479	3.295
**100K**	CMAPLE	-2,010,053	-55	**10.021**	1	**1.825**	1
	MAPLE	**-2,009,998**	0	18.631	1.859	6.199	3.397
**200K**	CMAPLE	**-3,425,616**	0	**19.236**	1	**3.569**	1
	MAPLE	-3,425,673	-57	58.094	3.020	11.476	3.215

Table S2. Runtimes, peak memory usage, and log-likelihoods of the trees inferred by CMAPLE executed with the high-performance memory allocator - jemalloc (CMAPLE) and without jemalloc (CMAPLE_wo_jemalloc). The tests were conducted on 5K, 10K, 50K, 100K, and 200K SARS-CoV-2 sequences. Each test was run three times, and the average results were reported.

#sequences	Versions	Log-Likelihood	Difference from the best	Runtimes (hours)	Time saving compared with CMAPLE_ wo_jemalloc	Peak memory usage (GB)	Ratio to the lowest peak memory usage
5K	CMAPLE	-240820.106	0.000	0.049	0.00%	0.129	1.000
	CMAPLE_ wo_jemalloc	-240820.106	0.000	0.049		0.129	1.000
10K	CMAPLE	-391548.681	-0.009	0.135	0.20%	0.244	1.000
	CMAPLE_ wo_jemalloc	-391548.672	0.000	0.136		0.244	1.000
50K	CMAPLE	-1274343.252	-0.003	2.036	0.22%	1.056	1.000
	CMAPLE_ wo_jemalloc	-1274343.249	0.000	2.040		1.056	1.000
100K	CMAPLE	-2010053.299	0.000	10.021	7.71%	1.825	1.000
	CMAPLE_ wo_jemalloc	-2010053.302	-0.003	10.859		1.824	1.000
200K	CMAPLE	-3425615.639	0.000	19.236	4.72%	3.569	1.001
	CMAPLE_ wo_jemalloc	-3425616.251	-0.612	20.189		3.567	1.000

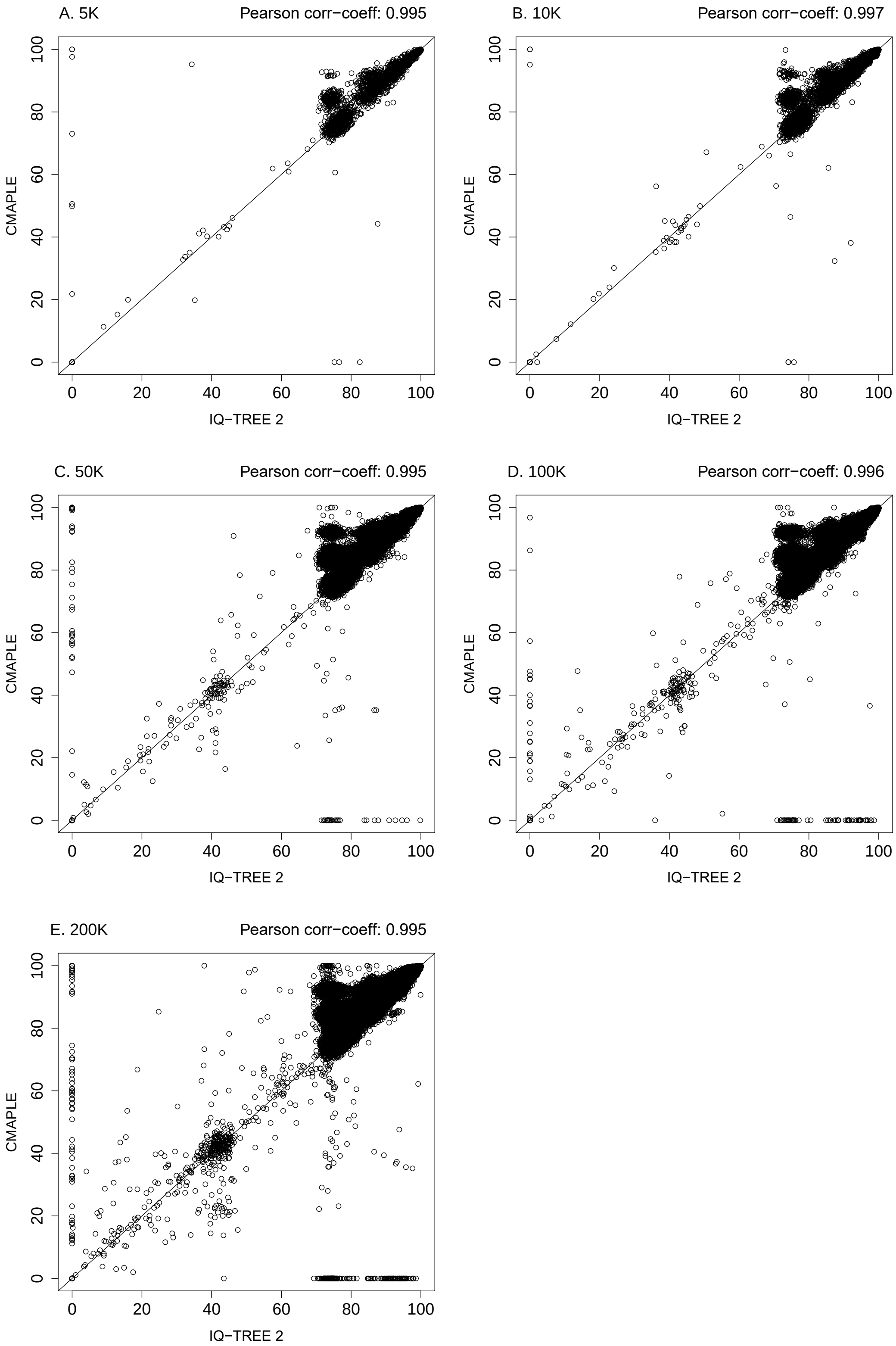

Figure S1. Branch supports computed by CMAPLE and IQ-TREE 2 of trees inferred by CMAPLE from 5K (sub-panel A), 10K (sub-panel B), 50K (sub-panel C), 100K (sub-panel D), and 200K (sub-panel E) real SARS-CoV-2 sequences.
